# *Salmonella gallinarum* strains from outbreaks of fowl typhoid fever in Southern Africa closely related to SG9R vaccines

**DOI:** 10.3389/fvets.2023.1191497

**Published:** 2023-07-05

**Authors:** Amanda Beylefeld, Celia Abolnik

**Affiliations:** Department of Production Animal Studies, Faculty of Veterinary Science, University of Pretoria, Pretoria, South Africa

**Keywords:** *Salmonella gallinarum*, fowl typhoid, reversion to virulence, SG9R, whole-genome comparison, vaccine

## Abstract

**Introduction:**

*Salmonella enterica* subspecies *enterica* serovar Gallinarum biovar Gallinarum (SG) is associated with fowl typhoid fever, and the attenuated rough strain SG9R is widely used as a vaccine in many regions. Reversion to virulence of vaccine strains was suspected as the cause during recent fowl typhoid fever outbreaks in poultry in South Africa and Eswatini.

**Methods:**

To compare nine field isolates with global wild-type SG9 strains and the two commercial SG9R vaccines in use, Nobilis^®^ SG9R and Cevac^®^-SG, we used whole-genome comparison with single-nucleotide polymorphism (SNP) detection.

**Results:**

SNP phylogenic analysis showed that all the southern African field isolates were more closely related to the vaccine strains than wild-type SG9 strains. Furthermore, SNPs in the pyruvate dehydrogenase (*ace*E) and/or lipopolysaccharide 1,2-glucosyltransferase (*rfa*J) genes, which are known markers of attenuation, were found in four of the field isolates along with intact *spv*, SPI-1, and SPI-2 gene clusters, providing conclusive evidence that these four isolates were originally vaccine strains that reverted to virulence. Five other field isolates lacked the SG9R attenuation markers, but variant analysis identified an SNP in the *yih*X gene, insertions in the *ybj*X and *hyd*H genes, and deletions in the *fts*K and *sad*A genes that were shared between the field isolates and vaccine strains but absent in wild-type SG9, indicating that these field isolates were also likely revertant vaccines.

**Discussion:**

Overall, this study highlights different mechanisms of reversion of two commercial vaccines, where virulence caused by field isolates closely related to the Nobilis^®^ SG9R vaccine was associated with the restoration of intact virulence gene clusters, and those derived from the Cevac^®^-SG vaccine were characterized by point mutations resulting in restored *ace*E and *rfa*J genes. A possible new marker of attenuation was identified as a point mutation in the *yih*X gene, as well as four new candidate genes that could potentially be used to distinguish current vaccine strains from wild-type strains using PCR assays.

## 1. Introduction

*Salmonella enterica* subspecies *enterica* serovar Gallinarum biovar Gallinarum (*S*. Gallinarum; SG) is associated with fowl typhoid fever, an acute or chronic septicemic disease, which leads to large economic losses in poultry due to reduced production and high mortality rates (1). SG is reported to be under control or eradicated in many developed countries, but it is still a common disease in many developing countries including South Africa (2). A recent review of the prevalence of SG between 1945 and 2021 revealed that SG is more prevalent in Europe and Africa, and *Salmonella enterica* subspecies *enterica* serovar Gallinarum biovar Pullorum (*S*. Pullorum; SP) is more prevalent in North and South America and Asia (2). There has also been an increase in the prevalence of both in the last decade (2). Clinical signs of infection include depression, anorexia, droopy wings, dehydration, and diarrhea (1). SG is a non-motile Gram-negative bacterium with slender rod morphology, appearing as small, smooth, blue-gray, or grayish-white colonies on standard beef agar (3). The size of the SG genome is between 4.2 and 4.9 megabase pair (Mbp) with an average GC content of 52%, 4,272 predicted coding sequences (CDSs), and seven rRNA operons (4). *S*. Gallinarum harbors a large virulent plasmid of 85 kilobase pair (kbp) and a small plasmid of 2.5 kbp, with the large plasmid encoding the *spvRABCD* virulence genes (5, 6).

Good management procedures can keep flocks free of SG and, along with eradication programs, have been used successfully to eradicate fowl typhoid in developed countries. However, the eradication of SG is unrealistic in developing countries including South Africa, where SG persists and is listed as a reportable and controlled animal disease (7). Antimicrobials used in the management of SG are ineffective in preventing and controlling the infection, with survivors eventually becoming asymptomatic carriers that perpetuate the persistence and spread of SG (8). Vaccination is common, and both inactivated and live attenuated vaccines have been developed, with the latter more widely used and the preferred method of disease control. Inactivated vaccines are whole-killed bacteria that can elicit a strong antibody response, but these types of vaccines do not elicit a cell-mediated response, resulting in failure to clear the pathogen from the host (9). A few subunit vaccines, which consist of single multiple defined antigens, have also been developed against other Salmonella species, but these vaccines have not yet been successful in providing protection and are still being investigated (10). Live-attenuated vaccines consist of live bacteria that contain mutations or deletions resulting in the loss of functions essential to the metabolism, host-survivability, or virulence of the pathogen. These types of vaccines can elicit both antibody and cell-mediated immune responses, but they can persist in the host and carry the risk of reversion to virulence (11). Three live vaccines are currently registered for use in South Africa, namely the Onderstepoort Biological Product (OBP) Fowl Typhoid vaccine which is an attenuated rough SG strain 5503; Nobilis^®^ SG9R produced by MSD Animal Health, and Cevac^®^ S. Gallinarum 1000D produced by Ceva Animal Health. The latter two vaccines are based on the attenuated rough SG strain 9R (SG9R) and are also the most widely used globally. Strain SG9R was developed in 1956; however, the molecular basis for its attenuation was not known at the time. It has been proven to be safe for use in adult chickens protecting against both mortality and colonization of the organs (12). There is currently no validated polymerase chain reaction (PCR) assay available to distinguish between the field and vaccine strains of SG (1), but a triplex PCR assay to differentiate between the two *Salmonella gallinarum* serovars, SG and SP, and SG9R is described (13). The *glg*C and *spe*C genes were used to differentiate between SG and SP, and single-nucleotide polymorphisms (SNPs) found in a hypothetical protein were used to differentiate SG9R (13, 14); however, this PCR method was not validated against the SG9 parental or wild-type SG9R strains (13).

Reversion to virulence of live SG vaccines is a concern in many countries and has been the subject of numerous studies since the advent of DNA sequencing. Kwon and Cho (15) used Sanger DNA sequencing to compare specific genes between the SG9R vaccine strain and SG9R-like field isolates from a fowl typhoid outbreak in Korea. A nonsense mutation in the lipopolysaccharide (*rfa*) gene cluster was found to be a likely attenuation and they hypothesized that any residual virulence in the vaccine was due to intact SPI-2 and *spv* genes (15). A comparative proteome and transcriptome analysis of the SG9R vaccine strain with two wild-type strains, 287/91 and 06Q110, found that the lack of the phage shock protein (PspA), an ABC transporter protein, and a flagellar component FliM, contributed to the avirulent nature of the vaccine strain and that the impaired expression of the SPI-1 or SPI-2 type III secretion system (TTSS) is a key factor (16). It was also reported that numerous mutations are required for reversion to virulence unless the mutation completely restored the regulation of the SPI-1 and SPI-2 gene clusters (16). Van Immerseel et al. (17) used whole-genome sequencing and SNP detection to show that the SG9R vaccine was closely related to a field isolate, MB4523, from a fowl typhoid outbreak in Belgium, specifically that two SNPs in the pyruvate dehydrogenase (*ace*E) and/or lipopolysaccharide 1,2-glucosyltransferase (*rfa*J) genes were the most likely cause of reversion to virulence (17). SNP detection and whole-genome comparison were similarly applied to determine that multiple fowl typhoid outbreaks in Brazil were caused by field strains and not by the reversion of the Nobilis^®^SG9R and Cevac^®^ SG9R vaccine strains (18).

There has been a concerning increase in anecdotal reports from the field in South Africa and the neighboring country of Eswatini of suspected reversion of the SG9R vaccines to virulence, especially among layer hens. Flocks vaccinated with SG9R vaccines have developed symptoms typically associated with fowl typhoid fever, especially in immune-compromised flocks co-infected with other pathogens. Such disease could stem from the residual virulence of the vaccine or reversion to virulence through a single-point mutation (19). Immune-compromised flock or flocks with nutrition deficiency allows the SG9R strain to proliferate resulting in vertical transmission and re-isolation during routine testing (15, 20). The recommended method of administration of the vaccine is subcutaneous for optimal protection, but it can be administered orally for 60% protection (21), and the application of SG vaccines in drinking water is a common practice in the local industry. In this study, whole-genome sequencing and comparative genomics with SNP detection were used to determine whether recent fowl typhoid outbreaks had been caused by wild-type field SG strains introduced into the poultry flock from external sources, or alternatively the reversion to virulence of attenuated commercial SG9R vaccines.

## 2. Materials and methods

### 2.1. Ethical clearance

Research approval for this study was obtained from the Department of Agricultural, Land Reform and Rural Development (DALRRD) permit no. 12/11/1/1/8 (1608 LH), and ethical approval was obtained from the Research and Animal Ethics Committees of the University of Pretoria, under project no. REC187-19.

### 2.2. Culture and DNA isolation

The field isolates used in this study (*n* = 9) were obtained from the NOSA Pty (Ltd) veterinary laboratory, Centurion, South Africa, which originally cultured and identified SG from the splenic material of chickens submitted by veterinarians for routine diagnosis, from poultry farms with a case history and where SG infection was suspected (Table 1). Flocks were immunized according to the manufacturers' recommendations, i.e., the Cevac^®^-SG vaccine initial vaccination was at 4 weeks followed by a second vaccination at 6 to 8 weeks, and the Nobilis^®^ SG9R vaccine was administered at 6 weeks followed by boosters at 12-week intervals. The two freeze-dried vaccine strains, Nobilis^®^ SG9R (batch no. A155AJ01) from MSD Animal Health Pty (Ltd) and Cevac^®^ S. Gallinarum 1000D (batch no. 044/21) from Ceva Animal Health Pty (Ltd), were purchased from the manufacturers and reconstituted by adding 10 ml of distilled water to each vial. Whole-genome sequencing data for two of the Nobilis^®^ SG9R vaccines, SG strain SG9Ra (PRJNA206379), and SG9Rb (PRJNA206380), were available from the National Center for Biotechnology Information (NCBI) (RRID:SCR_006472) (22), but these sequences were generated from vaccine batches in 2001 and 2009, respectively; therefore, it was decided to sequence more recent and locally sourced batches of the vaccines.

**Table 1:** *Salmonella gallinarum* field isolates analyzed in this study.

**Isolate ID**	**Bird age**	**Region**	**Vaccine used**
SG_424947	36 weeks	Gauteng Province, South Africa	Cevac^®^-SG
SG_467574	Unknown	Gauteng Province, South Africa	Cevac^®^-SG
SG_462889	42 weeks	Mpumalanga Province, South Africa	Cevac^®^-SG
SG_432755	12 weeks	KwaZulu-Natal Province, South Africa	Cevac^®^-SG
SG_440297	65 weeks	Eswatini	Cevac^®^-SG
SG_447025	12 weeks	Gauteng Province, South Africa	Nobilis^®^ SG9R
SG_434265	Unknown	Gauteng Province, South Africa	Nobilis^®^ SG9R
SG_445509	12 weeks	North West Province, South Africa	Nobilis^®^ SG9R
SG_442695	Unknown	Gauteng Province, South Africa	Nobilis^®^ SG9R

To verify purity, the field isolates and vaccine strains were cultured on Columbia blood agar with 5% horse serum and MacConkey agar without crystal violet for 24 h at 37°C at the Bacteriology laboratory of the Department of Veterinary Tropical Diseases, University of Pretoria. All isolates were confirmed to be pure and were subsequently inoculated into 100 ml of brain–heart infusion (BHI) broth and propagated for 24 h at 37°C. Cells were pelleted from 1.5 ml of cultures using centrifugation (10,000 x g at 4°C for 10 min). Then genomic DNA was isolated using the PureLink^®^ Genomic DNA Kit (Invitrogen) according to the manufacturer's instructions.

### 2.3. Genome sequencing and assembly

The isolated genomic DNA was submitted to the Central Analytical Facility, University of Stellenbosch for Ion Torrent Sequencing on the Ion S5 sequencer at 200 x coverage. The quality of the read data was evaluated using FastQC (RRID:SCR_014583) (23), followed by read trimming using the trimming tool in CLC Genomics Workbench version 8.5.1 (CLC Bio-Qiagen, Aarhus, Denmark). The reads were *de novo* assembled in CLC Genomics Workbench using the default parameters with a 200 bp cutoff size for the contigs, and the resulting contigs were submitted to the public databases for molecular typing and microbial genome diversity (PubMLST) Species ID tool (RRID:SCR_012955) (24, 25). The quality of the whole-genome assemblies was assessed using the Quality Assessment Tool for Genome Assemblies (QUAST) (RRID:SCR_001228) (26, 27).

### 2.4. Phylogenetic analysis

Ion Torrent sequence reads data produced in this study as well as Illumina sequencing reads data of the 2001 (SRR1045124) and 2009 (SRR1045125) Nobilis^®^ SG9R vaccine were assembled to the reference SG strain 287/91 (NC_011274.1) using CLC Genomic Workbench version 8.5.1. All complete SG genomes available from the NCBI database (https://www.ncbi.nlm.nih.gov/data-hub/genome, accessed: 30 September 2022) were downloaded, namely *S*. Gallinarum strain 287/91, SG9 (NZ_CM001153), 07Q015 (NZ_CP077760), SCPM-O-B-4493 (NZ_CP088134), SCPM-O-B-4548 (NZ_CP088142), and ATCC9184 (NZ_CP019035) (18). *Salmonella enterica* subsp. *enterica* serovar Typhimurium strain LT2 (*S*. Typhimurium) (NC_003197) was used as the outgroup. The phylogenetic and molecular evolution (PhaME) analysis tool was used to extract the SNPs between the isolates, vaccine strains, and all available complete *S*. Gallinarum reference genomes (28). The PhaME pipeline also constructed a phylogeny using IQ-Tree (RRID:SCR_017254) with automatic model selection and ultrafast bootstrap with 1000 iterations. The consensus maximum likelihood tree was viewed in FigTree Version 1.4.4 (RRID:SCR_008515) (29–31).

### 2.5. SNP analysis

The sequencing read data generated in this study and sequencing reads data of the 2001 Nobilis^®^ SG9R vaccine (SRR1045124) were uploaded to the Galaxy web platform using the public server at usegalaxy.org (RRID:SCR_006281) (32). The rapid haploid variant calling and core genome alignment tool (Snippy) version 4.6.0 with default parameters was then used for variant analysis using SG strain 9 (NZ_CM001153) and its plasmid (CM001154) as the reference (33). Default parameters include a minimum coverage of 10 reads per position and a minimum of 90% of reads must differ from the reference to be considered a variant.

### 2.6. Whole-genome comparison

*De novo* assembled genomes were submitted to the RAST server and annotated (RRID:SCR_014606) (34). The Mauve Contig Mover (MCM) tool of the Multiple Genome Alignment (Mauve) program was used to align and sort the order of the contigs compared to the SG strain SG9 reference genome (RRID:SCR_012852) (35, 36). The reordered contigs were then used to perform a progressive Mauve alignment.

## 3. Results

### 3.1. Genome sequencing and assembly

DNA from nine SG isolates and two vaccine strains was sequenced, and the complete consensus genomes were *de novo* assembled. Ion torrent whole-genome sequencing produced good quality reads between 230 and 372 x coverage and 50 to 51% GC content. Using the *de novo* assembled contigs in PubMLST, all the isolates were confirmed as SG. The isolates' consensus genomes covered between 99.12 and 99.74 % of the reference strain (Table 2). A reference genome coverage of 100% is ideal, but factors such as sequencing errors resulting in difficulties with repetitive homopolymer regions, AT-rich region, a common problem in Ion Torrent sequencing, as well as gene repeats, insertions, and deletions could affect this value. Another measure of the quality of an assembly is the benchmarking universal single-copy ortholog (BUSCO) score that considers the number of highly conserved genes present or absent. For both vaccines and seven of the isolates, > 95% of the core genes could be identified and were therefore considered good assemblies. The remaining two isolates, SG_432755 and SG_440297, were considered adequate assemblies with 94.59% and 93.92% of the core genes identified, respectively.

**Table 2:** Sequencing and *de novo* assembly results.

**Isolate ID**	**Read information**	***De novo*** **assembly**						
	**% GC**	**Read count**	**Average length**	**Coverage**	**No. of contigs** >**200 bp**	**N50**	**% Ref covered**†	**BUSCO%**
SG_424947	51	8,908,409	194.33	372	163	138,573	99.40	95.95
SG_445509	51	8,561,519	192.36	354	146	118,970	99.12	94.59
SG_467574	51	6,894,508	187.53	278	131	131,256	99.41	95.27
SG_462889	51	5,613,246	190.95	230	140	142,678	99.74	96.62
SG_442695	51	7,897,635	191.25	324	145	118,855	99.28	95.95
SG_432755	51	7,667,885	189.4	312	160	117,786	99.43	94.59
SG_447025	51	7,464,820	193.65	310	218	116,430	99.19	95.27
SG_434265	51	7,271,775	194.97	304	208	137,266	99.32	95.27
SG_440297	50	71,59,445	192.43	296	140	11,1641	99.42	93.92
Nobilis^®^ SG9R	51	7,318,088	190.83	300	173	11,8666	99.46	95.27
Cevac^®^-SG	50	8,502,499	189.14	345	155	141,528	99.23	95.27 (2.03)

**†**Reference: *S*. Gallinarum strain 287/91 (NC_011274.1).

### 3.2. Phylogenetic analysis

**Figure 1:**
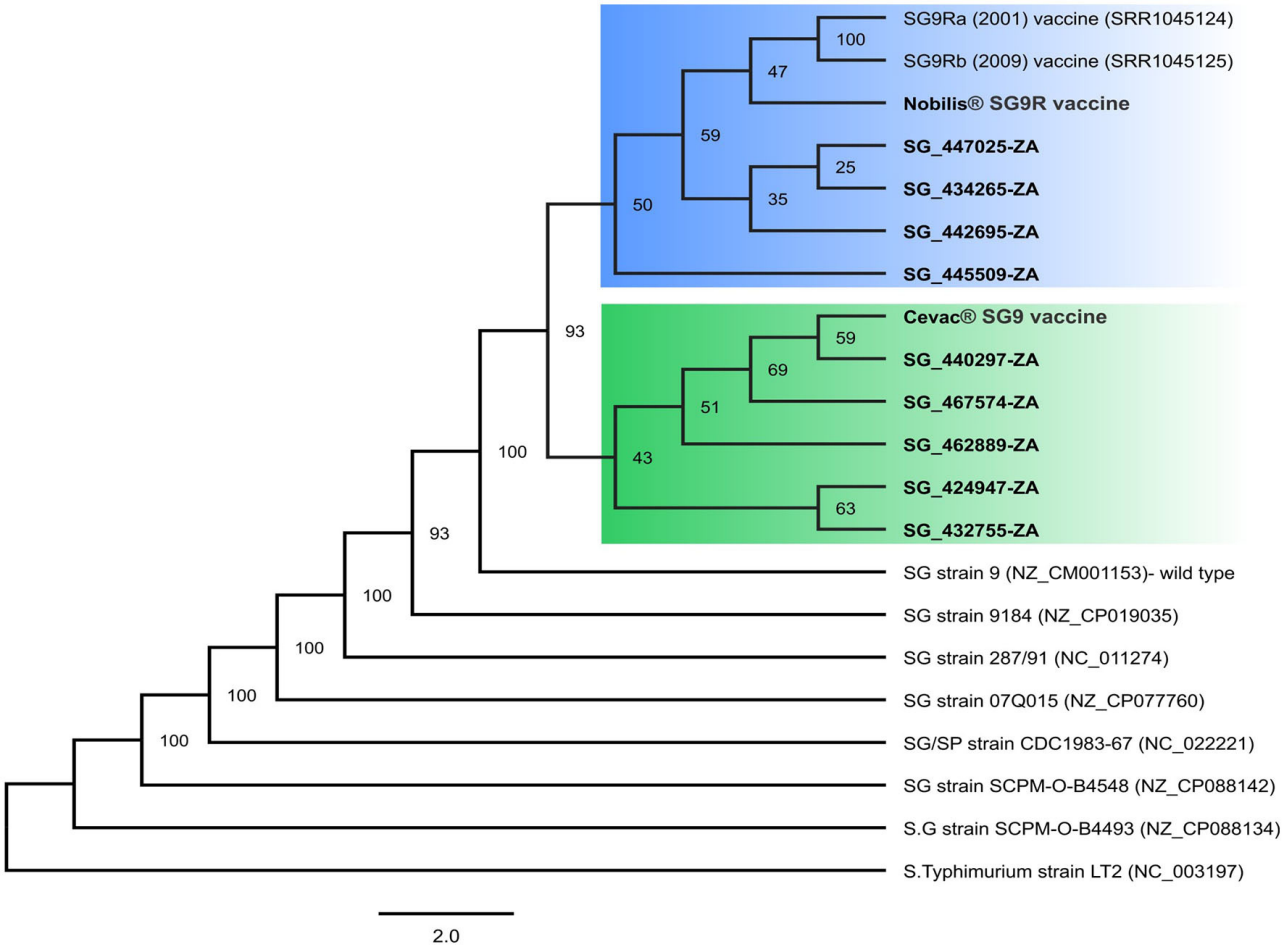
SNP-based maximum likelihood phylogeny of complete SG genomes. Thousand bootstrap trees were constructed with bootstrap support (%), shown at the nodes. Sequences generated in this study are highlighted in boldface. Two distinct clades related to the Nobilis^®^SG9R and Cevac^®^SG9 vaccines are highlighted in blue and green, respectively.

A maximum likelihood SNP phylogenetic tree was used to infer the relationship of the isolates to available complete SG reference genomes (Figure 1). As expected, the vaccines were closely related to the parental SG9 vaccine strain (100% bootstrap support), but all the isolates were also closely related to the SG9 wild-type strain (100% bootstrap support). The isolates also formed two distinct clades with 93% bootstrap support. Isolates SG_440297, SG_467575, SG_462889, SG_424947, and SG_432755 formed a clade with the Cevac^®^-SG vaccine; and SG_442695, SG_447025, SG_434265, and SG_445509 formed a clade with the Nobilis^®^ SG9R vaccine, which is consistent with the vaccines used in those flocks. The 2001 and 2009 Nobilis^®^ SG9R vaccines grouped more closely with the newly sequenced Nobilis^®^SG9R vaccine as expected, but a low bootstrap support (59%) was obtained. The low bootstrap value could be the result of improvements in sequencing technologies or more likely that some natural mutations occurred over time during the production of this vaccine, but no data for the production method were available in the public domain. The 2001 and 2009 Nobilis^®^ SG9R strains were previously found to be identical (17); therefore, only the 2001 sequence was used for further analysis.

### 3.3. SNP analysis

An SNP analysis using wild-type strain SG9 as a reference revealed that the Cevac^®^ SG vaccine strain had 20 variations (6 deletions, 9 insertions, and 5 SNPs) in total. The SNPs included the previously described SNPs associated with attenuation, i.e., point mutations in the *ace*E gene resulting in an amino acid change and the *rfa*J gene resulting in a premature stop codon (Table 3). Isolate SG__424947 with 21 variations (6 deletions, 8 insertion, and 7 SNPs), SG_467574 with 18 variations (6 deletions, 7 insertions, and 5 SNPs), SG_462889 with 22 variations (5 deletions, 8 insertions, and 9 SNPs), SG6_432755 with 18 variations (5 deletions, 8 insertions, and 5 SNPs), and SG_440297 with 17 variations (6 deletions, 7 insertions, and 4 SNPs) lacked the two characteristic point mutations in *ace*E and *rfa*J, but shared other variations with the vaccine strains. The Cevac^®^ SG vaccine shared three SNPs with these five isolates in the intergenic region (IGR) upstream from the *gol*B gene and missense mutations in the *yih*X and *msc*M genes.

**Table 3:** Core SNP analysis of southern African isolates and the vaccines compared to *S*. Gallinarum strain SG9 (NZ_CM001153).

**Strain SG9 genome**			**SNP**		**Cevac^^®^^ SG**	**SG_4 24947**	**SG_4** **67574**	**SG_4 62889**	**SG_4** **32755**	**SG_4 40297**	**Nobilis^^®^^ SG9R (2001)**	**Nobilis^^®^^ SG9R (2021)**	**SG_4** **45509**	**SG_4 42695**	**SG_4** **47025**	**SG_4 34265**
**Position**	**Codon (aa)**†	**Gene (strand)**	**Codon (aa)**†	**Mutation effect**												
177,092	**A**CC (Thr)	*pdh*R (+)	**G**CC (Ala)	Missense	-	-	-	**✓**	-	-	-	-	-	-	-	-
178684	AT**G** (Met)	*ace*E (+)	AT**A** (Ile)	Missense	-	**✓**	-	-	-	-	-	-	-	-	-	-
179,676	A**T**C (Ile)	*ace*E‡ (+)	A**A**C (Asn)	Missense	**✓**	-	-	-	-	-	**✓**	**✓**	**✓**	**✓**	**✓**	**✓**
376,015	T	IGR	C		-	**✓**	-	-	-	-	-	-	-	-	-	-
399,350	G	IGR	A		**✓**	**✓**	**✓**	**✓**	**✓**	**✓**	-	-	-	-	-	-
529,551	**C**AG (Gln)	*acr*B (-)	**T**AG (stop)	Nonsense	-	-	-	-	-	-	-	**✓**	-	-	-	-
613,223	A**T**C (Ile)	PMR (-)	A**C**C (Thr)	Missense	-	-	-	**✓**	-	-	-	-	-	-	-	-
797,047	T	IGR	C		-	-	-	-	-	-	**✓**		-	-	-	-
132,6985	C	IGR	A		-	**✓**	-	-	-	-	-	-	-	-	-	-
1,411,839	G**A**T (Asp)	*cha*B (+)	G**G**T (Gly)	Missense	-	-	-	**✓**	-	-	-	-	-	-	-	-
1,494,998	C**T**G (Leu)	*sap*B (-)	C**G**G (Arg)	Missense	-	-	-	-	**✓**	-	-	-	-	-	-	-
1,897,456	A**G**G (Arg)	*pnc*A (+)	A**A**G (Lys)	Missense	-	-	-	-	**✓**	-	-	-	-	-	-	-
2,039,050	**T**CT (Ser)	*csg*C (-)	**T**CC (Ser)	Synonymous	-	-	-	**✓**	-	-	-	-	-	-	-	-
2,20,1836	GC**T** (Ala)	*asm*A (-)	GC**C** (Ala)	Synonymous	-	-	-	-	-	**✓**	-	-	-	-	-	-
2,274,601	G	IGR	A		-	-	**✓**	-	-	-	-	-	-	-	-	-
2,380,479	**G**AA (Glu)	*sse*L (+)	**A**AA (Lys)	Missense	-	-	-	**✓**	-	-	-	-	-	-	-	-
2,639,881	G**G**C (Gly)	*sin*I (-)	G**A**C (Asp)	Missense	-	-	-	**✓**	-	-	-	-	-	-	-	-
2,928,432	G	IGR	C								**✓**					
2,978,572	G**A**C (Asp)	*stf*D (+)	G**C**C (Ala)	Missense	-	-	-	-	-	-	-	**✓**	-	-	-	-
3,432,990	TA**C** (Tyr)	*btu*B (-)	TA**A** (stop)	Nonsense	-	-	-	-	-	-	**✓**	-	-	-	-	-
3,548,690	**G**GA (Gly)	*rbn* (-)	TC**A** (stop)	Nonsense	-	-	-	-	-	-	**✓**	-	-	-	-	-
3,548,702	**C**TG (Leu)	*rbn* (-)	**A**TG (Met)	Missense	-	-	-	-	-	-	**✓**	-	-	-	-	-
3,549,508	**T**AC (Tyr)	*yih*X (-)	**C**AC (His)	Missense	**✓**	**✓**	**✓**	**✓**	**✓**	**✓**	**✓**	**✓**	**✓**	**✓**	**✓**	**✓**
3,834,254	AC**C** (Thr)	*uhp*B (+)	AC**T** (Thr)	Synonymous	-	-	**✓**	-	-	-	-	-	-	-	-	-
3,904,243	T**C**A (Ser)	*rfa*J‡ (+)	T**A**A (stop)	Nonsense	**✓**	-	-	-	-	-	**✓**	**✓**	**✓**	**✓**	**✓**	**✓**
4,219,765	C	IGR	T		-	**✓**	-	-	-	-	-	-	-	-	-	-
4,422,925	G**A**T (Asp)	*msc*M (-)	G**G**T (Ala)	Missense	**✓**	**✓**	**✓**	**✓**	**✓**	**✓**	-	-	-	-	-	-
4,555,602	**A**GG (Pro)	*yji*K (+)	**G**GG (Pro)	Synonymous							**✓**					

Aa, amino acid; ✓, detected;, not detected.

^†^Indicates the codon position of interest with the position of interest highlighted in bold.

^‡^SNPs identified in previous publications as important attenuation mechanisms for the SG9R vaccines; PMR, putative membrane region; IGR, intergenic region.

Vaccine strain Nobilis^®^ SG9R (2001) and the newly sequenced Nobilis^®^ SG9R had 14 (5, deletions and 9 SNPs) and 19 (6 deletions, 8 insertions, and 5 SNPs) sequence variations, respectively. Both vaccines contained the aforementioned characteristic point mutations in the *ace*E and *rfa*J genes and only shared one other SNP in the *yih*X gene. SG_445509 with 16 variations (5 deletions, 8 insertions, and 3 SNPs), SG_442695 with 18 variations (6 deletions, 9 insertions, and 3 SNPs), SG_447025 with 17 variations (6 deletions, 8 insertions, and 3 SNPs), and SG_434265 with 17 variations (6 deletions, 8 insertions, and 3 SNPs) also contained both aforementioned point mutations in the *ace*E and *rfa*J genes. All four isolates shared only one additional SNP with the vaccine strains in the *yih*X gene. Two other unique SNPs in the *acr*B and *stf* D genes were observed in the more recent Nobilis^®^ SG9R vaccine.

The Cevac^®^ SG vaccine had 11 insertions and deletions (indels) in common with the 5 isolates that were grouped in the same clade. Only three of the indels were in genes, i.e., a frameshift insertion in the *ybj*X, a disruptive insertion in the *hyd*H gene, and a disruptive deletion in the *sad*A gene (Table 4). All three of these indels were also found in the isolates that formed part of the Nobilis vaccine clade. The new Nobilis^®^ SG9R vaccine shared two additional deletions with the isolates that were in the same clade, i.e., a disruptive deletion in the *kef* C gene and a conservative deletion in the *fts*K gene. The deletion in the *kef* C gene was also found in the other isolates, but not in the Cevac^®^ SG vaccine, and the deletion in the *fts*K gene was found in the Cevac^®^ SG vaccine and only two other isolates (SG_467574 and SG_440297). The Nobilis^®^ SG9R (2001) vaccine strain only had five deletions, of which only one was in the same location as all the isolates and other vaccine strains, and it is in an intergenic region (IGR).

**Table 4:** Insertion and deletion variations in vaccine strains, southern African isolates, and the *S*. Gallinarum strain compared with SG9 reference strain (NZ_CM001153) and its plasmid (CM001154).

**Strain SG9 genome**			**Mutation**	**Cevac^^®^^ SG9R**	**SG_4 24947**	**SG_4 67574**	**SG_4 62889**	**SG_4 32755**	**SG_4 40297**	**Nobilis^^®^^ SG9R (2001)**	**Nobilis^^®^^ SG9R (new)**	**SG_4 45509**	**SG_4 42695**	**SG_4 47025**	**SG_4 34265**
**Position**	**Gene (Strand)**	**Type**	**Effect**												
100,069	*kef*C (+)	del	Disruptive deletion	-	**✓**	**✓**	**✓**	**✓**	**✓**	-	**✓**	**✓**	**✓**	**✓**	**✓**
197,279	IGR	del		**✓**	**✓**	**✓**	**✓**	**✓**	**✓**	**✓**	**✓**	**✓**	**✓**	**✓**	**✓**
600,352	IGR	ins		**✓**	**✓**	-	-	**✓**	-	-	**✓**	-	**✓**	**✓**	-
602,471	IGR	ins		**✓**	**✓**	**✓**	**✓**	**✓**	**✓**	-	**✓**	**✓**	**✓**	**✓**	**✓**
810,194	*oad*B2 (+)	ins	Disruptive insertion	-	-	-	-	-	-	-	-	**✓**	**✓**	-	**✓**
951,764	*ybj*X (-)	ins	Frameshift	**✓**	**✓**	**✓**	**✓**	**✓**	**✓**	-	**✓**	**✓**	**✓**	**✓**	**✓**
972,791	*fts*K (+)	del	Conservative deletion	**✓**	-	**✓**	-	-	**✓**	-	**✓**	**✓**	**✓**	**✓**	**✓**
1,114,959	PMR	del	Frameshift	-	-	-	-	-	-	**✓**	-	-	-	-	-
1,809,143	IGR	del		-	-	-	-	-	-	**✓**	-	-	-	-	-
1,857,660	IGR	ins		**✓**	**✓**	**✓**	**✓**	**✓**	**✓**	-	**✓**	**✓**	**✓**	**✓**	**✓**
1,940,779	IGR	del		-	-	-	-	-	-	**✓**					
2,495,665	IGR	del		**✓**	**✓**	**✓**	**✓**	**✓**	**✓**	-	**✓**	-	**✓**	**✓**	**✓**
2,928,428	IGR	del		**✓**	**✓**	**✓**	**✓**	**✓**	**✓**	-	**✓**	**✓**	**✓**	**✓**	**✓**
3,195,273	*ygi*R (-)	del	Disruptive deletion	**✓**	-	-	-	-	-	-	-	-	-	-	-
3,596,603	*hyd*H (-)	ins	Disruptive insertion	**✓**	**✓**	**✓**	**✓**	**✓**	**✓**	-	**✓**	**✓**	**✓**	**✓**	**✓**
3,821,715	*dsd*C (+)	del	Frameshift	-	**✓**	-	-	-	-	-	-	-	-	-	-
3,933,393	*sad*A (-)	del	Disruptive deletion	**✓**	**✓**	**✓**	**✓**	**✓**	**✓**	**✓**	**✓**	**✓**	**✓**	**✓**	**✓**
4,005,523	IGR	ins		**✓**	**✓**	**✓**	**✓**	**✓**	**✓**	-	**✓**	**✓**	**✓**	**✓**	**✓**
4,278,436	*yjb*G (+)	ins	Frameshift & stop	**✓**	-	-	**✓**	-	-	-	-	-	-	-	-
4,453,593	IGR	ins		**✓**	**✓**	**✓**	**✓**	**✓**	**✓**	-	**✓**	**✓**	**✓**	**✓**	**✓**
4,509,854	IGR	ins		**✓**	**✓**	**✓**	**✓**	**✓**	**✓**	-	**✓**	**✓**	**✓**	**✓**	**✓**
**Strain SG9 plasmid**														
21,447	*spv*B (+)	del	Disruptive deletion	**✓**	**✓**	**✓**	**✓**	**✓**	**✓**	-	**✓**	**✓**	**✓**	**✓**	**✓**

✓, detected; -, not detected; PMR, putative membrane region; IGR, intergenic region.

An SNP analysis of the plasmid revealed only a single disruptive deletion in the polyproline linker (PPL), a proline repeat region, of the *spv*B gene but it was present in all the vaccines and isolates. The plasmid sequence data for Nobilis^®^ SG9R (2001) were not available (Table 4).

### 3.4. Whole-genome comparison

**Figure 2:**
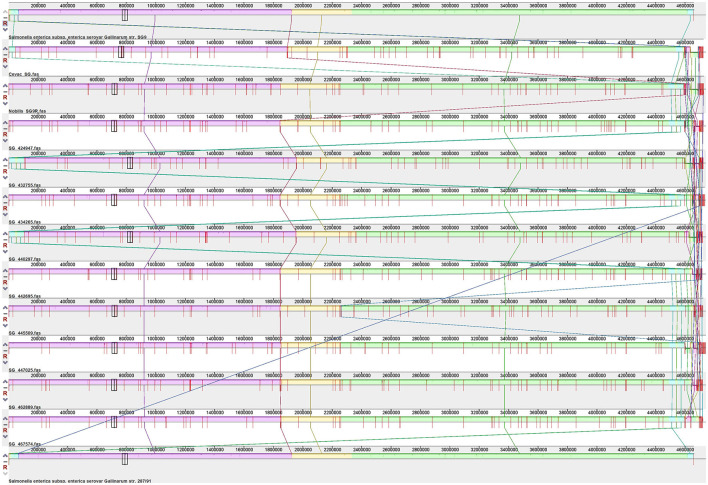
Alignment *S*. Gallinarum strain 9, SG9R vaccine strains, isolates, and *S*. Gallinarum strain 287/91. All the genomes were aligned with progressive Mauve using the default parameters. Similarly colored LCB blocks indicate homologous regions and lines linking these regions between the genomes.

A comparative genome alignment progressive Mauve produced nine locally collinear blocks (LCBs) (Figure 2). Full synteny was observed between the isolates, vaccines, and SG strain 9, with small areas of genome rearrangement observed near the 5′end of the genome. The reference genome had a similar arrangement, but the contig overlapping this region was split and arranged to preserve the genome coordinates compared to the closely related reference SG strain 287/91 (37).

## 4. Discussion

The strain SG9R is the most widely used SG vaccine strain in the world and one of the only two strains registered for use as vaccines in South Africa to prevent fowl typhoid fever. Reversion of the vaccine strain has been studied previously with varying results. In this study, we used an approach similar to that of Van Immerseel et al. (17), combining comparative whole-genome analysis with SNP detection to investigate the possible reversion to the virulence of vaccines during outbreaks of fowl typhoid fever in South Africa and Eswatini in 2017. SNP-based maximum likelihood phylogeny analysis showed that all the local field isolates were more closely related to the vaccine and SG9 strain than any of the other available SG reference strain genomes.

The SNP analysis revealed the anticipated known markers of attenuation in the SG9R vaccine strains. A missense point mutation in the second position of a codon encoding an isoleucine (Ile) resulted in an amino acid change to asparagine (Asn) in the pyruvate dehydrogenase (*ace*E) gene and a nonsense mutation in the second codon in the *rfa*J gene at position 3,904,243 from a serine (Ser) to a stop codon. However, whether the mutation in the *ace*E gene affects the function of the pyruvate dehydrogenase E1 component is unknown (13). The premature stop codon in the *ace*E gene which encodes a lipopolysaccharide (LPS) 1,2,-glucosyltransferase could result in a truncated LPS core and loss of the O-antigen side chain (15, 38). A missense point mutation in the *yih*X gene found in all vaccines resulted in a tyrosine (Tyr) to histidine (His) amino acid change. The *yih*X gene encodes glucose-1-phosphatase, which is a putative enzyme with an unknown function in SG, but possibly plays a role in metabolism and is upregulated by the PhoP/Q system, that controls the expression of numerous genes involved in the virulence and survival of Gram-negative bacteria (39).

Van Immerseel et al. (17) previously reported no changes between the 2001 and 2009 Nobilis^®^-SG9R vaccines and only identified four SNPs in total, in the *ace*E, *rfa*J, *btu*B, and *gal*T genes. Only the first three were located using the Snippy pipeline in this study, which could be attributed to the different technologies used. A comparison of the 2001 and newly sequenced Nobilis^®^-SG9R vaccines demonstrated that the genome of the vaccine has changed slightly over the past two decades, but the older and newer vaccines were still more closely related to each other than to the field isolates. The more recent Nobilis^®^-SG9R vaccine also contained substantially more insertions and deletions compared to the 2001 Nobilis^®^-SG9R vaccine, but most of these were in the IGR with one disruptive deletion in the *sad*A gene. The *sad*A gene encodes a trimeric autotransporter adhesin and plays a role in biofilm formation and adhesion in *S. typhimurium*. However, a loss in this gene does not cause a significant effect on infection (40). This deletion was also observed in the Cevac^®^ SG vaccine, and all the isolates tested in this study and could be a potential marker to distinguish between vaccine- and wild-type strains.

The four isolates, SG_445509, SG_442695, SG_447025, and SG_434265, that were closely related to the Nobilis^®^-SG9R vaccine contained both known markers of attenuation in *ace*E and *rfa*J genes, a point mutation in the *yih*X gene, plus intact spv, SPI-1 and SPI-2 gene clusters (data not shown), and as such these are revertant vaccines and most likely the cause of the outbreaks observed on the respective farms (15). The remaining five isolates were closely related to the Cevac^®^ SG vaccine, but none of these isolates contained the known attenuation markers. However, these isolates were closely related to and formed a distinct clade with the Cevac^®^ SG vaccine. Point mutations resulting in intact *ace*E and *rfa*J genes along with intact spv, SPI-1, and SPI-2 gene clusters were most likely the reason for the virulence observed in these flocks. Van Immerseel et al. (17) similarly reported that these mutations were the likely cause of the reversion to virulence observed in Belgium. These isolates furthermore shared a point mutation in the *yih*X gene, and one additional missense mutation in the *msc*M gene was found in all the isolates and the Cevac^®^ SG vaccine. The *msc*M gene encodes a miniconductance mechanosensitive channel and plays a role in regulating osmotic pressure (41). This protein is not well-characterized in Salmonella, and the effect of mutations remains unknown (42).

Analysis of the indels revealed two insertions and one deletion shared between all the isolates and vaccines sequenced in this study. A frameshift insertion was identified in the *ybj*X (also known as *som*A) gene, which encodes a virK homolog protein and plays a minor role in virulence in *Salmonella enterica* serovar Enteritidis (SE) by modulating membrane proteins, affecting bacterial motility, secretion, and altering the membrane proteins to evade host adaptive immune response (43, 44). Mckelvey et al. (43) also demonstrated that this gene plays a role in SE persistence in chickens. SG is a non-motile pathogen; therefore, its motility would not be affected, but mutations in this gene could affect the ability of SG to evade the host immune response as has been shown in SE (43). A disruptive insertion was found among all the isolates sequenced in this study in the *hyd*H gene, which encodes the sensor kinase of the two-component zinc resistance-associated regulator (ZraS) and possibly plays a role in *Salmonella* infection, acting as a signal (45). Even though neither of these insertions was observed in the 2001 Nobilis^®^-SG9R vaccine, it is an interesting discovery that warrants further investigation to determine if these are markers of attenuation in the newer vaccine strain and to differentiate vaccine from wild-type strains.

A disruptive deletion in the *kef* C gene was observed in all the isolates and the new Nobilis^®^-SG9R vaccine, but not in the Cevac^®^ SG vaccine. This gene encodes the glutathione-regulated potassium-efflux system protein KefC which controls the efflux of potassium and protects against electrophiles in *Escherichia coli* (46). Little information is available on this gene in *Salmonella* including any effects mutations may have. A conservative deletion in the *fts*K gene was found in the Cevac^®^ SG vaccine, and only two isolates were closely related to it, as well as in the Nobilis^®^-SG9R vaccine and all the isolates related to it. The *fts*K encodes DNA translocase FtsK and plays a role in chromosome segregation, but Wang et al. (47) found that indels in this gene probably have no effect on the protein function.

The *spv*B virulence plasmid gene encodes Mono (ADP-ribosyl) transferase SpvB and inhibits phagocyte function when it is secreted into the macrophage cytoplasm (15). Both of the vaccines and all the isolates contained the expected lenght of nine prolines in the PPL. The length of the PPL is linked to the increased virulence, thus, even if the PPL is only nine proline residues long, it is still intact and the strain can still show diminished virulence, along with the intact SPI-1 and SPI-2 type III secretion system (48).

There are some limitations in this study that could be addressed in future research. Previous studies, like that of Van Immerseel et al. (17) and De Carli et al. (17) used genomic comparison with SNP detection and phylogenetics to show how field strains were related to the SG9R vaccine and this study also focused only on similar genomic methods. However, the virulence of the field strains was not studied *in vivo* and would be advantageous in future studies to confirm that observed SG symptoms were due to the reversion of the vaccine to virulence and not due to possible residual virulence of the vaccine.

In conclusion, all the field isolates from a recent spate of fowl typhoid outbreaks in South Africa and Eswatini were closely related to the SG9R vaccine strains in use, and since no wild-type strains were identified, reversion to virulence of the vaccine is the most likely cause of these outbreaks. Reversion to virulence in the Nobilis^®^-SG9R vaccine was associated with intact known virulence factors, spv, SPI-1, and SPI-2, whereas the virulence of Cevac^®^-SG vaccine-derived revertants was likely associated with point mutations resulting in intact *ace*E and *rfa*J genes. The latter findings still require *in vivo* verification, as does the cause driving the consistent selection of these specific point mutations. Additional markers identified in this study, i.e., an SNP in the *yih*X gene, insertions in the *ybj*X and *hyd*H genes, and a deletion in the *sad*A gene could, with further investigation, prove to be useful in distinguishing current SG9R vaccines from wild-type strains by targeted PCR assays.

## Data availability statement

The data presented in the study are deposited in the NCBI repository (https://www.ncbi.nlm.nih.gov/genbank/), accession numbers CP118112–CP118133 and SRR23450481–SRR23450491.

## Ethics statement

Ethical approval was obtained from the Research and Animal Ethics Committees of the University of Pretoria, under project no. REC187-19.

## Author contributions

AB and CA contributed to the conception and design of the study. AB organized the database, performed the bioinformatic analyses, and wrote the first draft of the manuscript. Both authors contributed to the manuscript revision, read, and approved the submitted version.
